# Influencing Factors of Knowledge, Attitude, and Practice regarding Medical Nutrition Therapy in Patients with Diabetes: A National Cross-Sectional Study in Urban China

**DOI:** 10.1155/2017/8948452

**Published:** 2017-08-16

**Authors:** Zijian Li, Haimin Jin, Wei Chen, Zilin Sun, Lulu Jing, Xiaohui Zhao, Sainan Zhu, Xiaohui Guo, China NEEDs Study Group

**Affiliations:** ^1^Department of Parenteral and Enteral Nutrition, Peking Union Medical College Hospital, Chinese Academy of Medical Sciences and Peking Union Medical College, Beijing, 100730, China; ^2^Department of Clinical Nutrition, Affiliated Hospital of Taishan Medical University, Taian, Shandong, China; ^3^Department of Endocrinology, Zhongda Hospital, Institute of Diabetes, Medical School, Southeast University, Nanjing, Jiangsu, China; ^4^Department of Clinical Nutrition, Peking University First Hospital, Beijing, China; ^5^Abbott Nutrition Research & Development, Shanghai, China; ^6^Statistics Office, Peking University First Hospital, Beijing, China; ^7^Department of Endocrinology, Peking University First Hospital, Beijing, China; ^8^Diabetes Care and Education Study Group, China Diabetes Society, Beijing, China

## Abstract

To investigate the knowledge-attitude-practice (KAP) score in diabetes patients living in urban China regarding Medical Nutrition Therapy (MNT) and explore the influencing factors, this national survey recruited diabetes and prediabetes patients in 40 hospitals across 26 provinces in China. A self-designed questionnaire was used to collect the data and assess the knowledge, attitude, and practice regarding MNT. Logistic regression was used to explore the factor influencing KAP scores. A total of 6441 diabetes patients (mean age: 60.02 ± 13.14 years) completed this survey. The mean glycosylated hemoglobin (HbA1c) level was 8.12 ± 2.12%, and the control rate of HbA1c (HbA1c < 7.0%) was 38.92%. Of the total, 53.56% had received MNT education. Over half of the patients had a poor total KAP score as well as poor K, A, and P scores. Patients with higher KAP scores had higher control rate of HbA1c (*P* < 0.05) but lower levels of fasting plasma glucose (FPG) and 2-hour postprandial blood glucose (2h-PG). Gender, occupation, residence, education level, and MNT education could influence the KAP scores (*P* < 0.05). This study showed that diabetes patients in urban China generally had poor understandings and practices related to MNT. Patients with higher KAP scores exhibited better control of blood glucose.

## 1. Introduction

Diabetes is a common chronic metabolic disease that greatly affects people's health and quality of life. It is estimated that about 415 million individuals aged 20–79 years lived with diabetes in 2015, while another 318 million were diagnosed with impaired glucose tolerance. In addition, the number of patients with diabetes aged 20–79 years is expected to increase to 642 million by 2040 [1]. With improvements in living standards and changes of life styles, more and more individuals are diagnosed with diabetes in developing countries. The incidence rate of diabetes in China was as high as 11.6% in 2013 [2]. In 2015, over 109.6 million individuals were living with diabetes in China, which made China the first leading country with the highest number of adult diabetes patients in the world. In addition, the medical cost of diabetes in China was estimated at over 51 billion US dollars [1]. A nationwide survey in patients with type 2 diabetes in 2010 showed that the control rate of blood glucose was only 32.18% (glycosylated hemoglobin [HbA1c] < 7.0%) [3]. Therefore, it is urgent to take effective measurements to prevent and manage diabetes in China [4]. Medical Nutrition Therapy (MNT), namely, applying special nutritional interventions for specific diseases in clinical practice, was first proposed by the American Diabetes Association (ADA) in 1994, which recommended that equal attention should be paid to nutrition therapy and drug therapy [5]. In 2006, the ADA urged that all diabetes patients should receive individualized MNT under the guidance of special clinicians or dietitians, which could help them achieve the ideal treatment target [6]. The* China Medical Nutrition Therapy Guideline for Diabetes* was issued in 2011 [7], which pointed out that MNT is the foundation for the treatment for diabetes. In 2013, a panel of experts from the area of endocrinology and nutrition developed and issued the first* China Expert Consensus of Medical Nutrition Therapy for Diabetes* [8], which again highlighted that MNT is the foundation of diabetes management. In addition, the consensus also pointed out that, for the blood glucose control in subjects with obesity, metabolic syndromes, prediabetes, diabetes, pregnancy with gestational hyperglycemia, and pregnancy in the perioperative period, MNT has several advantages including higher feasibility, safety, and effectiveness and thus could improve the prognosis and reduce the medical expenses. Therefore, MNT has already been considered as an essential tool for the management of diabetes. Previous studies have already shown that applying MNT in diabetes management can substantially improve the rate of blood glucose control [9, 10]. Furthermore, individualized MNT, according to the specific conditions of each patient, could further bring benefits to both medical and economic aspects [11].

MNT has already been applied in China for several years and received extensive attention from health care professionals. However, the application of this tool is still not optimal, due to its dependence on the communications between clinicians and patients and requirement of high cooperation of the patients. Several studies have already investigated the application of MNT in Chinese diabetes patients in recent years [12–14]. However, the sample sizes of all these studies are relatively low, and thus the results still need to be validated. In addition, the knowledge, attitude, and practice regarding MNT, as well as the factors that could affect the application of MNT in China are still unclear. Therefore, we first conducted this national survey to assess the knowledge, attitude, and practice regarding MNT among diabetes patients in urban China and to explore the influencing factors.

## 2. Materials and Methods

### 2.1. Study Design

A multicenter cross-sectional study was conducted by the Diabetes Care and Education Study Group of the Chinese Diabetes Society in 40 tertiary, secondary, and community hospitals across 26 cities in China. Fixed-point continuous sampling was adopted to recruit patients with diabetes and prediabetes. The patients were mainly included from the outpatient department, while the ones from the inpatient department accounted for no less than 10% of all the patients included.

### 2.2. Patients

The inclusion criteria for the patients were as follows: (1) age ≥ 18 years; (2) being diagnosed with impaired glucose tolerance or diabetes for 1 year or more or being with gestational diabetes mellitus; (3) consciousness and ability to correctly understand and respond to the questions; and (4) willingness to participate in the study and signing informed consent form. Patients with one or more of the following items were excluded: (1) age < 18 years; (2) inability to complete the survey due to physical or psychological disorders; (3) refusing to participate in the survey; (4) refusing to sign the informed consent form; and (5) being ineligible due to other conditions assessed by the investigators.

### 2.3. Questionnaire Design

A structured questionnaire was developed by a panel of experts consisting of clinicians, nurses, dieticians, and other investigators. Each question in the questionnaire was developed from clinical practices and was closely associated with current MNT education in China. The questionnaire was discussed and modified several times before being applied in the survey. The preliminary survey was conducted before the initiation of this study to help modify the questionnaire and improve the validity and reliability of the questionnaire. The Cronbach's alpha values were 0.679, 0.717, 0.412, and 0.440 for the total KAP score, K score, A score, and P score, respectively.

The questionnaire contains two parts. The first part of the questionnaire collects the demographic and disease information, as well as physical examination data, including gender, age, height, weight, occupation, education level, residence, blood glucose level, and duration and type of diabetes. The second part of the questionnaire includes the questions on KAP regarding MNT (Appendix). Knowledge (K) of MNT is assessed by 6 questions (Q4, Q5, Q8, Q9, Q10, and Q15), attitude (A) is assessed by 3 questions (Q11, Q12, and Q20), and practice (P) is assessed by 10 questions (Q2, Q3, Q6, Q7, Q13, Q14, Q16, Q17, Q18, and Q19). Question 1 is an independent question, which assesses whether the patient had been educated about MNT before. For the questions Q11, Q12, Q13, Q14, Q19, and Q20, 1 point is obtained when the answer is “No,” while for the other questions, 1 point is obtained when the answer is “Yes.” The scores for K, A, and P are obtained by adding the scores of all the questions in each section, and the highest scores possible are 6, 3, and 10, respectively. The total score for the questionnaire is 19, which is calculated by adding the scores of K, A, and P together. The higher score indicates that the patient has better KAP regarding MNT.

The total KAP score and the scores of K, A, and P are considered poor when the score is less than or equal to the median value (the median values for total KAP, K, A, and P score were 9, 3, 1, and 5, resp.).

### 2.4. Data Collection and Measurements

The questionnaire survey was conducted by trained investigators (clinicians, education nurses, or dieticians) assigned by each of the participating hospitals, through face-to-face interviews with the patients.

Physical examinations were performed by experienced nurses. The following data were collected during the physical examinations: height, weight, waist circumference, hip circumference, systolic blood pressure, diastolic blood pressure, fasting plasma glucose (FPG), 2-hour postprandial blood glucose (2h-PG), and glycosylated hemoglobin (HbA1c). All these data were measured by standard methods that have been widely accepted and applied in clinical practice within 3 months prior to the study initiation.

### 2.5. Quality Control

Over 6000 patients were included in this study to provide a sufficient sample size. Strict quality control was applied throughout the processes of data collection and processing. The investigators were uniformly trained several times before the study to minimize the bias and improve the validity and reliability of the questionnaire. Repetitive checks were executed by special investigators after the questionnaires were completed. In addition, double entry and validation of the data were performed within 72 h after the questionnaires were completed.

### 2.6. Ethical Considerations

This study was approved by the Ethics Committee of Peking University First Hospital and was performed strictly according to the Declaration of Helsinki. All the participants were informed of the contents and other essential information, and written informed consent forms were obtained from all patients before the survey was started. This study was also registered in the Chinese Clinical Trial Registry (number ChiCTR-OCS-14005204).

### 2.7. Statistical Analyses

SAS 9.2 (SAS 9.2; SAS Institute Inc., Cary, NC, USA) was used for the statistical analyses in this study. Quantitative data are described with means ± standard deviation (SD), while qualitative data are described as frequencies and percentages. Independent* t*-test or Wilcoxon rank-sum test was used for the comparisons of quantitative data between two groups, and analysis of variance (ANOVA) or nonparametric test was used for the comparisons of quantitative data among three or more groups. Chi-square test was used for the comparisons of qualitative data among different groups. Multivariate linear regression was used to explore the factors influencing the MNT score. All the statistical analyses were two-sided, and *P* < 0.05 was considered statistically significant.

## 3. Results

### 3.1. General Characteristics

Between May 18, 2014, and August 22, 2014, 6932 patients were recruited in this study, and 6441 completed questionnaires were obtained after deleting those with missing data or logical errors, yielding an effective rate of 92.9%. Importantly, 94.67% of these patients had type 2 diabetes, while only 5.32% of the patients had type 1 diabetes, prediabetes, or other types of diabetes. Among these patients, 3061 were females and 3380 were males, and their mean age was 60.02 ± 13.14 years; 37.98% of the patients were over 65 years of age. The mean disease duration was 9.36 ± 7.22 years, and 62.04% of the patients had the disease for a duration of 1–10 years. The mean body mass index (BMI) of the patients was 24.76 ± 3.67 kg/m^2^, and 56.67% of the patients were overweight or obese (BMI ≥ 24 kg/m^2^). Only 18.00% of the patients had an education level of college or higher. 58.42% of the patients were retired, and 53.56% of the patients had been educated about MNT before (Table 1).

The mean FBG of the patients was 8.15 ± 2.91 mmol/L, and 21.2% of the patients had aFBG level lower than 6.1 mmol/L. The mean 2h-PG of the patients was 11.78 ± 4.28 mmol/L, and 12.1% of the patients had a 2h-PG lower than 7.8 mmol/L. The mean HbA1c of the patients was 8.12 ± 2.12%, and the control rate among them was 38.92% (HbA1c < 7.0%).

### 3.2. KAP Score

A relatively poor KAP score was obtained from 54.54% of the patients. In addition, 62.79%, 61.85%, and 56.16% of the patients had poor K, A, and P scores in this study (Table 2).

### 3.3. Association between Total KAP Score and Blood Glucose Control

**Figure 1:**
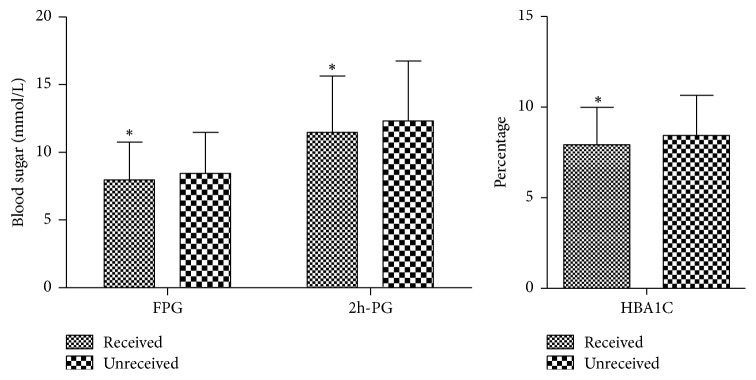
*Influence of the MNT education on blood glucose levels.* ^*∗*^Significant difference compared to the unreceived MNT group, *P* < 0.001.

The FPG, 2h-PG, and HbA1c levels in the patients educated about MNT before were significantly lower than those in patients not educated before (Figure 1). In addition, the FPG, 2h-PG, and HbA1c levels in the patients with total KAP scores of 10–19 were significantly lower than in the ones with the scores of 0–9 (Table 3).

### 3.4. Influencing Factors of KAP Score

Chi-square test showed that total KAP scores of the patients were influenced by gender, BMI, education level, occupation, residence, and MNT education (Table 4). The total KAP scores were significantly higher in female patients than in male patients (OR, 1.24; 95% confidence interval [CI], 1.10–1.40; *P* = 0.001), higher in patients with a BMI < 24 kg/m^2^ than in those with a BMI ≥ 24 kg/m^2^ (OR, 1.17; 95% CI, 1.05–1.31; *P* = 0.007), higher in patients with senior high school (OR, 1.65; 95% CI, 1.42–1.91; *P* < 0.001), junior college (OR, 2.30; 95% CI, 1.95–2.72; *P* < 0.001), or undergraduate education level or above (OR, 2.92; 95% CI, 2.47–3.45; *P* < 0.001) compared with those of individuals with a junior middle education level or below, higher in the patients who had retired than in the ones in service (OR, 1.55; 95% CI, 1.34–1.79; *P* < 0.001), and higher in those who received MNT education than in those who did not (OR, 5.06; 95% CI, 4.50–5.69; *P* < 0.001). However, no significant association between disease duration and total KAP score was found (Table 5).

Consistent with the total KAP score, the results of this study also showed that the K scores were significantly higher in patients with a BMI < 24 kg/m^2^ than in those with a BMI ≥ 24 (OR, 1.21; 95% CI, 1.08–1.37; *P* = 0.001); higher in patients with senior high school (OR, 1.50; 95% CI, 1.29–1.75; *P* < 0.001), junior college (OR, 2.11; 95% CI, 1.78–2.50; *P* < 0.001), or undergraduate education level or above (OR, 2.58; 95% CI, 2.18–3.05; *P* < 0.001) than in those with a junior middle education level or below; higher in retired patients than in the ones in service (OR, 1.51; 95% CI, 1.30–1.75; *P* < 0.001); and higher in patients who received MNT education than in those who did not (OR, 5.45; 95% CI, 4.81–6.17; *P* < 0.001). However, gender, age, and disease duration were not significantly associated with the K score (Table 5).

The A score in this study was significantly associated with only gender, education level, occupation, and MNT education. In detail, the A score was significantly higher in female patients than in the male patients (OR, 0.86; 95% CI, 0.77–0.96; *P* = 0.008). However, the education level was positively associated with the A score. Compared with the patients with a junior middle education level or below, patients with senior high school (OR, 1.22; 95% CI, 1.06–1.41; *P* = 0.005) and undergraduate education level or above (OR, 1.25; 95% CI, 1.07–1.45; *P* = 0.005) had significantly higher A scores. In addition, the A score was also significantly higher in retired patients than in in-service patients (OR, 1.35; 95% CI, 1.17–1.54; *P* < 0.001). However, in contrast to total KAP score and K score, patients who received MNT education had significantly lower A scores than those who did not (OR, 0.87; 95% CI, 0.78–0.97; *P* = 0.013; Table 5).

The P score in this study was significantly influenced by gender, education level, occupation, place of residence, and MNT education. The P score in female patients was significantly higher than that in male patients (OR, 1.23; 95% CI, 1.10–1.39; *P* < 0.001). In agreement with the total KAP score, K score, and A score, education level was also positively associated with the P score. Compared with the patients with a junior middle education level or below, those with senior high school (OR, 1.40; 95% CI, 1.21–1.62; *P* < 0.001), junior college (OR, 2.12; 95% CI, 1.81–2.50; *P* < 0.001), and undergraduate education level or above (OR, 2.65; 95% CI, 2.26–3.12; *P* < 0.001) had significantly higher A scores. In addition, patients who were retired (OR, 1.44; 95% CI, 1.25–1.66; *P* < 0.001) and who received MNT education (OR, 3.45; 95% CI, 3.08–3.86; *P* < 0.001) had significantly higher P scores than those in service and did not receive MNT education, respectively (Table 5).

## 4. Discussion

To our knowledge, this is the first large-scale study investigating the application of MNT and the influencing factors in patients with diabetes living in urban China. In this national cross-sectional survey with over 6000 patients included, the results showed that over half of the patients with diabetes in urban China received MNT education. However, the knowledge and practice of MNT in the patients with diabetes were suboptimal, and the total KAP score as well as K, A, and P scores was poor in more than half of the patients.

A previous study in China has shown that patients with diabetes had positive attitudes but relatively poor nutrition knowledge and practices [16], which has been confirmed in another study in the patients with diabetes in South Africa [17]. However, these findings were not in agreement with our results. Both the previous studies only included limited patients (162 and 217 patients, resp.); thus, the results could be easily biased. In addition, the questionnaires used in those two studies were not identical to the one used in our study, which could also contribute to the differences with our findings. The K score of the patients with diabetes in this study was slightly higher than the score reported in a previous study in Malaysia [9], suggesting that Chinese patients may have a higher level of MNT knowledge than those in Malaysia. However, the disease duration in our study was evidently longer (9.33 ± 7.13 years versus 6.3 ± 4.9 years). The agony from the disease in such long duration could effectively promote the patients to search for related knowledge and actively seek help from clinicians. The attitudes and practices of MNT were not reported in the Malaysia study, and thus we could not compare the findings of these two studies.

Both individualized and group MNT could effectively improve the control of blood glucose in patients with diabetes. Previous studies showed that after the application of MNT the FPG, postprandial blood glucose, and HbA1c levels all decreased significantly [9, 10]. A study in China also showed that, after 1-year education about MNT in patients with type 2 diabetes, the FPG level was significantly lower than the level before the education [14]. For patients with prediabetes, individualized MNT is effective at reducing the HbA1c level compared with usual care [18]. These findings suggested that improving the knowledge and practice of MNT in patients with diabetes could substantially improve the blood glucose control. In our study, the results showed that blood glucose levels were negatively associated with the KAP scores. Patients with higher KAP scores had lower FPG, 2h-PG, and HbAc1 levels. In addition, the HbAc1 control rate also increased significantly with the KAP score, which was in agreement with previous studies [9, 16]. The mean HbAc1 level in this study was 8.12 ± 2.12%, while the control rate was only 38.92%, which was higher than that reported in a national study in patients with type 2 diabetes performed in 2010 (32.18%) [3]. The increase could be associated with the scale-up of comprehensive treatment and self-management of diabetes in China in recent years. However, the control rate in this study was still lower than the results reported in Korea, USA, European countries, and Japan [19, 20]. These findings show that although MNT has been applied in China for about 5 years, the knowledge and practice of MNT in diabetes patients in urban China are still suboptimal, and the controlling rate of blood glucose is still very low.

Many factors could affect the knowledge, attitude, and practice of MNT in patients with diabetes. The findings in this study showed that the total KAP score as well as the K, A, and P scores of the patients was significantly associated with gender, occupation, education level, residence, and MNT education. A previous study showed that female patients tend to have higher total KAP scores and K and P scores but lower A scores than male patients, suggesting that although male patients have more active attitude to MNT than female patients, the practice is still lagging behind.

The findings in this study showed that in-service patients had lower total KAP scores as well as lower K, A, and P scores than retired patients. We speculated that this could be associated with the fact that the in-service patients still have careers to worry about, and thus their work and accompanying social activities restricted them from remaining concerned about the disease conditions [21]. In addition, social activities in China are generally accompanied by banquets, which could further impair the MNT of in-service patients. In contrast, retired patients are past the busiest stages in life generally, and they have enough time and interest to learn the knowledge about diabetes management. The findings in this study also showed that patients who lived in rural areas had significantly lower KAP scores than those who lived in urban areas, which was in agreement with previous findings [22, 23]. This could be associated with the fact that most patients in rural areas had a relatively low education level, which restricted them from clearly and correctly understanding the knowledge about MNT. The findings that patients with lower education level had lower KAP scores also supported this hypothesis. In addition, patients from rural areas generally had a lower income; therefore, they had a heavier work load and mental stress than the ones from urban areas, which not only further restricts them from obtaining MNT-related knowledge, but also impairs their practices in MNT [24]. However, another study in China showed no significant differences in the knowledge, attitude, and practice scores regarding MNT between the diabetes patients from rural and urban areas; however, that study did find that the patients with longer disease duration had higher total KAP score and practice score [16]. The difference from our findings could be associated with the sample size, source of subjects, and the questionnaires used.

The findings in this study showed that the total KAP scores as well as the K, A, and P scores in patients who received MNT education were significantly higher than those in the patients who did not, which is in agreement with the results reported in previous studies [14, 25, 26], suggesting that providing MNT education for diabetes patients could not only improve the related knowledge but also help improve the attitudes and practices of applying MNT and thus finally improving the self-management of blood glucose level.

### 4.1. Limitations of the Study

As a cross-sectional study, this study could not clarify the causal relationship between the factors and KAP scores on MNT. In addition, the retrospective method for collecting data could also introduce recall bias. However, this study could still provide valuable evidence for helping us to understand the factors influencing the application of MNT in Chinese diabetes patients and thus further provide evidence for further clinical studies and practices. To minimize the bias, investigators were uniformly trained before the survey started. In addition, the preliminary survey, as well as strict quality control in this study, could also help minimize, although not prevent, the bias. This study used a self-designed questionnaire to collect the data, and the Cronbach's alpha values for A and P scores were relatively low. However, the previous adoption of the same questionnaire by many other researchers in China suggests that this questionnaire can reflect well the MNT attitude and behaviors of diabetes patients. Therefore, this questionnaire was still used in our study despite the relatively low alpha values for A and P scores.

In conclusion, this study showed that although over half of the patients with diabetes living in urban China had received MNT education, over half of the patients still had poor understanding and practices of MNT, and the control rate of blood glucose was still very low. Patients with higher KAP scores were with better control of blood glucose. These findings suggest that MNT education is critical for patients with diabetes. However, the method of MNT education should be different, according to gender, BMI, occupation, and education level of the patients.

## Figures and Tables

**Table 1:** General characteristics of the patients.

Category	Subcategory	Data
Total number		6441

Age (y)		60.20 ± 13.14
	18–65, *n* (%)	3995 (62.02)
	65-, *n* (%)	2446 (37.98)

Gender	Female, *n* (%)	3061 (47.53)
	Male, *n* (%)	3380 (52.47)

BMI (kg/m^2^)^*∗*^		24.76 ± 3.67
	<24, *n* (%)	2791 (43.33)
	24-, *n* (%)	3650 (56.67)

Duration (y)		9.60 ± 7.22
	1–10, *n* (%)	3995 (62.04)
	10-, *n* (%)	2444 (37.96)

Educational status	Middle school or below, *n* (%)	2690 (41.76)
	Senior high school, *n* (%)	1517 (23.55)
	Junior college, *n* (%)	1075 (16.69)
	Undergraduate or above, *n* (%)	1159 (18.00)

Occupation	Retiree, *n* (%)	3763 (58.42)
	In service, *n* (%)	2005 (31.13)
	Others, *n* (%)	690 (10.71)

Diagnosis	Type 1 diabetes, *n* (%)	161 (2.50)
	Type 2 diabetes, *n* (%)	6098 (94.67)
	Other types of diabetes, *n* (%)	33 (0.51)
	Prediabetes, *n* (%)	149 (2.31)

MNT education experience	Yes, *n* (%)	3450 (53.56)
	No, *n* (%)	2991 (46.44)

FBG (mmol/L)		8.15 ± 2.91
	<6.1^*∗*^ (%)	21.2

2h-PG (mmol/L)		11.78 ± 4.28
	<7.8^*∗*^ (%)	12.1

HbA1c (%)		8.12 ± 2.12
	<7.0^*∗*^ (%)	38.92

BP (mmHg)		130.25 ± 16.89/77.54 ± 10. 3
	<140/90^*∗*^ (%)	77.35

^*∗*^The reference values for HbA1c and the classification of BMI were according to the directions of “China Guideline For Type 2 Diabetes” (2013) [15].

**Table 2:** Distribution of the MNT, knowledge, attitude, and practice scores.

Category	Subcategory	Frequency (*n*)	Percentage (%)
KAP score	0–9 (poor)	3513	54.54
	10–19 (good)	2928	45.46
Knowledge score	0–3 (poor)	4044	62.79
	4–6 (good)	2397	37.21
Attitude score	0-1 (negative)	3984	61.85
	2-3 (positive)	2457	38.15
Practice score	0–5 (poor)	3617	56.16
	6–10 (good)	2824	43.84

**Table 3:** Comparison of FBG, 2h-PG, and HbA1c between KAP score groups.

KAP score	FPG		2h-PG		HbA1c	
	Mean ± SD	CR^‡^	Mean ± SD	CR^‡^	Mean ± SD	CR^‡^
0–9	8.58 ± 3.16	19.58	12.66 ± 4.65	11.07	8.48 ± 2.21	31.85
10–19	7.69 ± 2.50^*∗*^	25.41	11.01 ± 3.90^*∗*^	17.48	7.77 ± 1.96^*∗*^	46.59

SD, standard deviation, mmol/L; CR, controlling rate (%); ^*∗*^*P* < 0.001 compared to the f KAP score group, 0–9; ^‡^*P* < 0.001 for significant difference determined by the *χ*^2^ test.

**Table 4:** Factors associated with MNT KAP, knowledge, attitude, and practice, scores [*n* (%)].

Category	KAP score *n* (%)		Knowledge score *n* (%)		Attitude score *n* (%)		Practice score *n* (%)	
	Good	Poor	Good	Poor	Good	Poor	Good	Poor
Gender								
Female	1447 (47.27)	1614 (52.73)	1155 (37.73)	1906 (62.27)	1116 (36.46)	1945 (63.54)	1389 (45.37)	1672 (54.63)
Male	1481 (43.82)	1899 (56.18)	1242 (36.75)	2138 (63.25)	1341 (39.67)	2039 (60.33)	1435 (42.46)	1945 (57.54)
	*X* ^2^ = 7.74	*P* = 0.005	*X* ^2^ = 0.67	*P* = 0.413	*X* ^2^ = 7.04	*P* = 0.008	*X* ^2^ = 5.57	*P* = 0.018

Age								
18–65	1702 (42.60)	2293 (57.40)	1418 (35.49)	2577 (64.51)	1491 (37.32)	2504 (62.68)	1677 (41.98)	2318 (58.02)
65-	1226 (50.12)	1220 (49.88)	979 (40.02)	1467 (59.98)	966 (39.49)	1480 (60.51)	1147 (46.89)	1299 (53.11)
	*X* ^2^ = 34.60	*P* = 0.000	*X* ^2^ = 13.33	*P* = 0.000	*X* ^2^ = 3.03	*P* = 0.082	*X* ^2^ = 14.89	*P* = 0.000

BMI								
<24	1326 (47.51)	1465 (52.49)	1100 (39.41)	1691 (60.59)	1041 (37.30)	1750 (62.70)	1266 (45.36)	1525 (54.64)
24-	1602 (43.89)	2048 (56.11)	1297 (35.53)	2353 (64.47)	1416 (38.79)	2234 (61.21)	1558 (42.68)	2092 (57.32)
	*X* ^2^ = 8.36	*P* = 0.004	*X* ^2^ = 10.18	*P* = 0.001	*X* ^2^ = 1.50	*P* = 0.221	*X* ^2^ = 4.60	*P* = 0.032

Duration (y)								
1–10	1727 (43.23)	2268 (56.77)	1406 (35.19)	2589 (64.81)	1500 (37.55)	2495 (62.45)	1716 (42.95)	2279 (57.05)
10-	1200 (49.10)	1244 (50.90)	990 (40.51)	1454 (59.49)	956 (39.12)	1488 (60.88)	1107 (45.29)	1337 (54.71)
	*X* ^2^ = 21.08	*P* = 0.000	*X* ^2^ = 18.32	*P* = 0.000	*X* ^2^ = 1.58	*P* = 0.21	*X* ^2^ = 3.38	*P* = 0.066

Education								
Middle school or below	948 (35.24)	1742 (64.76)	768 (28.55)	1922 (71.45)	958 (35.61)	1732 (64.39)	936 (34.80)	1754 (65.20)
Senior high school	719 (47.40)	798 (52.60)	579 (38.17)	938 (61.83)	618 (40.74)	899 (59.26)	664 (43.77)	853 (56.23)
Junior college	590 (54.88)	485 (45.12)	489 (45.49)	586 (54.51)	421 (39.16)	654 (60.84)	574 (53.40)	501 (46.60)
Undergraduate or above	671 (57.89)	488 (42.11)	561 (48.40)	598 (51.60)	460 (39.69)	699 (60.31)	650 (56.08)	509 (43.92)
	*X* ^2^ = 226.36	*P* = 0.000	*X* ^2^ = 180.62	*P* = 0.000	*X* ^2^ = 13.27	*P* = 0.004	*X* ^2^ = 199.80	*P* = 0.000

Occupation								
Retiree	1888 (50.40)	1858 (49.60)	1535 (40.98)	2211 (59.02)	1508 (40.26)	2238 (59.74)	1782 (47.57)	1904 (50.83)
In service	814 (40.60)	1191 (59.40)	673 (33.57)	1332 (66.43)	704 (35.11)	1301 (64.89)	809 (40.35)	1196 (59.65)
	*X* ^2^ = 50.37	*P* = 0.000	*X* ^2^ = 30.33	*P* = 0.000	*X* ^2^ = 14.60	*P* = 0.000	*X* ^2^ = 27.51	*P* = 0.000

MNT education								
Yes	2181 (63.22)	1269 (36.78)	1874 (54.32)	1576 (45.68)	1293 (37.48)	2157 (62.52)	1988 (57.62)	1462 (42.38)
No	747 (24.97)	2244 (75.03)	523 (17.49)	2468 (82.51)	1164 (38.92)	1827 (61.08)	836 (27.95)	2155 (72.05)
	*X* ^2^ = 945.00	*P* = 0.000	*X* ^2^ = 930.22	*P* = 0.000	*X* ^2^ = 1.41	*P* = 0.236	*X* ^2^ = 572.91	*P* = 0.000

**Table 5:** Association of the factors with MNT knowledge, attitude, and practice scores.

Category	KAP score		Knowledge score		Attitude score		Practice score	
	OR (95.0% CI)	*P*	OR (95.0% CI)	*P*	OR (95.0% CI)	*P*	OR (95.0% CI)	*P*
*Gender*								
Male	1.00		1.00		1.00		1.00	
Female	1.24 (1.10–1.40)	0.001	1.08 (0.96–1.22)	0.212	0.86 (0.77–0.96)	0.008	1.23 (1.10–1.39)	0.000

*Age (y)*								
18–65	1.00		1.00		1.00		1.00	
>65	1.02 (0.89–1.17)	0.744	0.92 (0.80–1.06)	0.260	0.99 (0.87–1.12)	0.833	0.99 (0.87–1.12)	0.872

*BMI*								
>24	1.00		1.00		1.00		1.00	
<24	1.17 (1.05–1.31)	0.007	1.21 (1.08–1.37)	0.001	0.95 (0.85–1.06)	0.341	1.12 (1.00–1.25)	0.050

*Duration (y)*								
10-	1.00		1.00		1.00		1.00	
1–10	1.03 (0.91–1.16)	0.689	1.03 (0.91–1.17)	0.639	1.02 (0.91–1.14)	0.718	0.92 (0.82–1.04)	0.164

*Education*								
Middle school or below	1.00		1.00		1.00		1.00	
Senior high school	1.65 (1.42–1.91)	0.000	1.50 (1.29–1.75)	0.000	1.22 (1.06–1.41)	0.005	1.40 (1.21–1.62)	0.000
Junior college	2.30 (1.95–2.72)	0.000	2.11 (1.78–2.50)	0.000	1.17 (1.00–1.36)	0.052	2.12 (1.81–2.50)	0.000
Undergraduate or above	2.92 (2.47–3.45)	0.000	2.58 (2.18–3.05)	0.000	1.25 (1.07–1.45)	0.005	2.65 (2.26–3.12)	0.000

*Occupation*								
In service	1.00		1.00		1.00		1.00	
Retiree	1.55 (1.34–1.79)	0.000	1.51 (1.30–1.75)	0.000	1.35 (1.17–1.54)	0.000	1.44 (1.25–1.66)	0.000

*MNT education *								
No	1.00		1.00		1.00		1.00	
Yes	5.06 (4.50–5.69)	0.000	5.45 (4.81–6.17)	0.000	0.87 (0.78–0.97)	0.013	3.45 (3.08–3.86)	0.000

**Table 6:** 

Item	Question
*Knowledge*	
Q4	Knows the dietary management of diabetes has been upgraded into MNT.
Q5	Knows that a lot of professional associations recommend MNT as the foundation of diabetes prevention and treatment both at home and abroad.
Q8	Understands and remembers MNT recommendations provided by HCPs.
Q9	Knows the total amount of daily food that should be consumed.
Q10	Knows how to allocate the daily intake of food groups.
Q15	Knows the food restrictions or other factors that may induce malnutrition.
*Attitude*	
Q11	Too frightened to take a meal (or reduce intake) because of concerns about increased post-prandial glycaemia
Q12	Too frightened to eat fruits and sweets because of concerns about increased blood glucose.
Q20	Feels distressed or difficult to adhere to self-management according to the MNT recommendations provided by HCPs (multiple choices). If “yes”, the main obstacles for acceptance or adherence to MNT is; (1) no chance to understand; (2) the content is too difficult to be understood; (3) the requirement is too high to adhere to.
*Practice*	
Q2	Correctly determined their body-weight group (refer to BMI, kg/m^2^, low weight ≤18.5; normal 18.6–23.9; overweight 24.0–27.9; obesity ≥28).
Q3	Can calculate the ideal body weight.
Q6	Routinely pay attention to the nutrition status.
Q7	Are routinely provided with MNT recommendations by doctors, clinical dietitians, or nurses.
Q13	Has experienced hypoglycemia due to irregular life style choices.
Q14	Has experienced between-meal hypoglycemia, bedtime hypoglycemia, or nocturnal hypoglycemia
Q16	Routinely follows the recommendations of doctors or clinical dietitians when arranging daily diet,
Q17	Routinely eats more vegetables than meat in order to control blood glucose.
Q18	Routinely increases the intake of snacks as a compensation of the reduction of meals or staple food recommendations.
Q19	Routinely unable to execute the MNT recommendations because of various reasons.

## Appendix

The questionnaire about the knowledge, attitude, and practices of patients with diabetes and prediabetes toward MNT is shown in Table 6.
